# The COVID-19 Status of Patients Is an Essential Determinant for Decision-Making by Radiation Oncologists: A European Survey

**DOI:** 10.7759/cureus.22842

**Published:** 2022-03-04

**Authors:** Selma Ben Mustapha, Paolo Simoni, Nadège Dubois, Nicolas Jansen, Ferenc Lakosi, Antonio Silva Mota, Sara Ramella, Philippe Coucke

**Affiliations:** 1 Radiation Oncology, University Hospital of Liège, Liège, BEL; 2 Radiology, Queen Fabiola Children's University Hospital, Brussels, BEL; 3 Rheumatology, University Hospital of Liège, Liège, BEL; 4 Deaprtment of Public Health, University of Liège, Liège, BEL; 5 Radiation Oncology, Institute of Diagnostic Imaging and Radiation Oncology, Kaposvár University, Kaposvár, HUN; 6 Radiation Oncology, Institut Godinot, Reims, FRA; 7 Radiation Oncology, Università Campus Bio-Medico di Roma, Rome, ITA

**Keywords:** covid-19, organization, departments, survey, radiotherapy

## Abstract

Aim: To assess the tendencies of radiation oncologists (ROs) in adjusting radiotherapy treatments (RTH) according to the coronavirus disease 2019 (COVID-19) status of patients during the early severe acute respiratory syndrome coronavirus 2 (SARS-COV2) pandemic in Europe.

Material and methods: An electronic survey was sent to 79 academic RTH departments across Europe. Only one respondent per institution was included. Respondents were asked how they would adjust RTH treatments based on COVID-19 status for more common cancers during the first wave of the pandemic. Respondents were also asked to report the number of external beam radiotherapy (EBRT) units and the number of new cases referred to their department. Descriptive statistical analysis was conducted focusing on different cancers.

Results: The overall response rate to the survey was 30.38% (24 institutions from 13 European countries). There was a wide range of different institutions regarding the number of patients, radiation oncologists, and facilities. A large proportion of respondents supported adjustment of RTH treatment (delay or switch to a shorter fractionation) for COVID-19-negative patients during the first wave of the pandemic only for early breast cancer (20% delay, 42.3% shorter), prostate cancer (53.6% delay, 21.4% shorter), and benign brain tumours (32% delay, 12% shorter). For COVID-19-negative patients with other cancers, most respondents recommended the standard RTH treatment. For COVID-19-positive patients, most respondents favoured a delay in RTH treatment or a shorter fractionation, regardless of cancer type and stage.

Conclusion: The patient's COVID status significantly influenced the decision to undergo RTH treatment, regardless of the type and aggressiveness of cancer.

## Introduction

The outbreak of coronavirus disease 2019 (COVID-19) in 2020 had a significant impact on cancer care management. Cancer patients are most vulnerable due to frequent contact with medical staff during treatment. In addition, cancer itself, current or previous treatments, and the age of cancer patients make them more likely to suffer from infectious diseases due to their weakened immune status [1,2].

For patients undergoing or planning to have radiotherapy, European Departments of Radiotherapy (RTH) have adapted their procedures to guarantee the best care for patients [3,4]. In addition, original articles, case reports, and editorials have been published during the pandemic addressing these issues in a broad or specific disease area or for a particular treatment modality [5-15]. However, recommendations to adapt RTH treatment according to the COVID-19 status of patients were only provided for head and neck tumours [13]. No recommendations were made to adjust RTH treatments for other cancer types depending on the patient's COVID status.

The American Society of Radiation Oncology (ASTRO) conducted a survey on more than 500 radiation oncologists (ROs) from various departments in the United States to assess the impact of this pandemic [3]. In Europe, the European Society of Radiotherapy and Oncology (ESTRO) has also published a survey to evaluate the adjustments that radiation oncology departments have had to make to treat patients safely [4]. Noticeably, these studies did not specifically focus on the impact of patients' COVID-19 status on RO decision-making regarding fractionation adjustment or delay of RTH treatment. In addition, these investigations did not clarify whether the impact of the COVID-19 status of the patient varied with the type of cancer and stage. 

The present survey aimed to assess the impact of the COVID-19 status of the patient on RTH treatment decision-making considering different cancer types and stages in academic hospitals around Europe.

## Materials and methods

Survey design

An electronic survey was designed to find out how European ROs planned to adapt RTH treatments, considering the COVID-19 status of patients and their cancer types and stages (Table 1).

**Table 1:** Tumour location and characteristics Her 2: Human epidermal growth factor receptor 2; NSCLC: non-small cell lung cancer; SBRT: stereotactic body radiotherapy

Tumour location or Brachytherapy indication	Tumour characteristics
Brain tumours	Glioblastoma, high-grade glioma (other than glioblastoma), low-grade glioma, benign brain tumour (grade 1 meningioma or acoustic neuroma), brain metastases
Head and neck cancer	
Lung cancer	NSCLC eligible for lung SBRT, locally advanced NSCLC
Gastrointestinal cancer	Oesophagal cancer, rectal cancer; both eligible for a radiotherapy treatment
Prostate cancer	Low-risk prostate cancer, favorable intermediate-risk prostate cancer, unfavorable intermediate-risk or high-risk prostate cancer, node-positive prostate cancer
Breast cancer	Ductal carcinoma in situ, numinal A/B N0M0, Her 2 enriched N0M0, triple-negative N0M0, luminal A/B N+M0, Her2 enriched or triple-negative N+M0
Brachytherapy indications	Cervical cancer, endometrial cancer, prostate cancer

A 25-item survey was developed in electronic format using the commercial software Survey-Monkey® (Momentive Inc., San Mateo, California, United States). The survey was built by the first author (SBM), covering the main cancer types, considering other clinically relevant characteristics when needed (e.g., presence of hormone receptors, risk profile, histology, stage). The first draft of the survey was sent to two academic ROs from different institutions and countries (FL Hungary, NJ Belgium). They were asked to assess the appropriateness and clarity of each item independently. Their comments and suggestions were included in the preliminary survey and validated in a consensus meeting. Finally, the survey was sent to the professional addresses of the respondents in April 2020. As the survey did not address patients or animals and no clinical experiments or records were included in this study, according to the declaration of Helsinki, ethics committee approval was waived.

Selection of respondents

Respondents were selected through a manual search of the Internet, looking for academic hospitals and ROs on national and European societies' websites. The search was performed to enrol at least one academic hospital from each European country. The survey was sent by email to all potential participants, with an automatic reminder to increase the response rate two weeks after the initial invitation.

Inclusion And Exclusion Criteria

The inclusion criteria were to be an academic RO actively involved in clinical activity in a European RTH department, to be available to provide personal information to be anonymised, and to consent that the data would be analysed and published anonymously.

The exclusion criteria were multiple respondents from the same institution (in the case of multiple respondents from the same institution, only the response of the senior RO was considered in the final analysis) and incomplete surveys.

Data collection

All survey responses were collected from April 13, 2020, to May 18, 2020. Survey data were extracted using the automated system of the electronic platform Survey-Monkey and exported to a Microsoft Excel worksheet (Microsoft Corp., Redmond, Washington, United States).

Statistical analysis

Descriptive statistics were performed for the number and countries of the respondents and for each type of cancer cumulating the collected data. For continuous values, we calculated the mean and range values.

## Results

Respondents' institutional features

We obtained a response rate of 30.38% (24 responses from 79 invitations). Responses were collected from 24 institutions in 13 European countries with an average of 1.84 participating institutions per country (Table 2).

**Table 2:** Number of respondents per country

Country	Respondents (24)
Spain	4
Germany	1
Italy	3
Portugal	3
Luxembourg	1
Netherlands	1
France	3
Slovenia	1
Slovakia	2
Hungary	1
Belgium	2
Ireland	1
Switzerland	1

The mean number of new patients treated per year was 2028, for a mean number of 10.4 senior ROs working in the department. The mean number of EBRT units was 4.5 (range 2-9). The mean number of brachytherapy devices was 1.3. Brachytherapy was not available in one institution. Out of 22 institutions, 17 (77.27%) did not have any IntraOperative RadioTherapy (IORT) devices (Table 3).

**Table 3:** Human resources and devices EBRT: external beam radiotherapy; IORT: intraoperative radiotherapy

Department features	Mean	Range
Number of new patients/years	2028	500-6200
Number of senior radiation oncologists	10.4	2-30
Number of EBRT devices	4.5	2-9
Number of brachytherapy devices	1.3	0-4
Number of IORT devices	0.2	0-1

RTH treatments proposed adjustments

**Figure 1:**
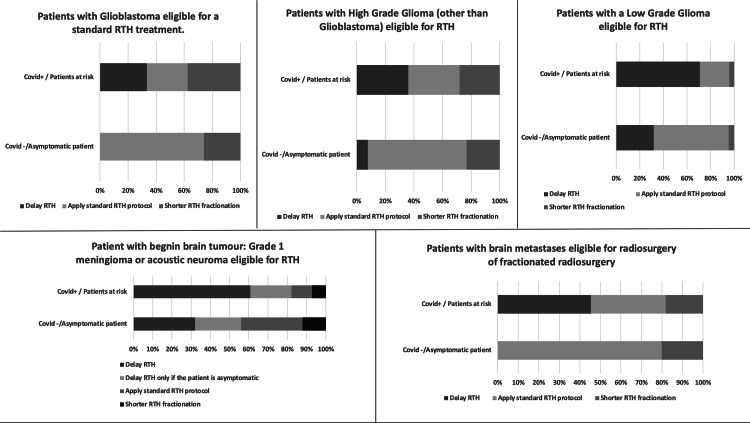
RTH treatment adaptations for different types of brain tumours according to the COVID-19 status of the patient RTH: radiotherapy; COVID-19: coronavirus disease 2019

The following results reflect what respondents would have done during the first wave of the COVID-19 pandemic period in Europe if they had had to deal with each radiotherapy indication proposed in the survey. Respondents were asked to consider that patients were eligible to receive an RTH treatment. In addition, we asked respondents to consider patients suspected to have COVID-19 infection as COVID-positive patients. Figure 1 shows the RTH treatment adaptations in the case of brain tumours.

According to respondents, among patients with glioblastoma eligible for RTH treatment, COVID-19-negative patients should have received the standard RTH protocol (80% of respondents) or a shorter RTH fractionation (20% of respondents). Respondents proposed no RTH treatment delay for this group of patients. COVID-19-positive patients should have received the standard RTH protocol according to 29.2% of respondents, a shorter fractionation according to 37.5% of respondents, and delayed according to 33.3% of respondents.

For high-grade gliomas, COVID-19-negative patients should have received the standard RTH protocol in 69.2% of respondents, a shorter RTH fractionation by 23.1% of respondents, and a delayed treatment according to 7.7% of respondents. COVID-19-positive patients with high-grade gliomas other than glioblastomas should have received the standard RTH protocol according to 36.0% of respondents, a shorter fractionation by 28% of respondents, and 36% of respondents recommended to delay RTH treatment.

In COVID-19-negative patients with low-grade gliomas, the standard RTH protocol was recommended by 63.6% of respondents, a shorter RTH fractionation was favoured by 4.5% of respondents, and 31.8% of respondents chose to delay RTH treatment. In COVID-19-positive patients with low-grade glioma, the standard RTH protocol was recommended in 25% of respondents, shorter fractionation was suggested in 4.2% of respondents, and 70.8% of respondents favoured a delay in RTH treatment.

For COVID-19-negative patients with brain metastases, the standard RTH protocol was suggested by 80% of respondents, a shorter RTH schedule was recommended in 20%, and no respondents were in favour of a delay in RTH treatment in this patient group. For COVID-19-positive patients, the standard RTH protocol was recommended by 36.3% of respondents, a shorter fractionation by 18.2%, and 45.5% of respondents favoured a delay in RTH treatment.

For patients with benign brain tumours including grade 1 meningioma or acoustic neuroma, 32% of respondents favoured delaying the RTH treatment for COVID-19-negative patients compared with 60.7% for COVID-19-positive patients. For asymptomatic benign brain lesions, for COVID-19-negative patients, 24% of respondents recommended delaying RTH treatment. For the same patients but with a positive COVID-19 status, a delay was suggested by 21.4% of respondents.

The standard RTH protocol was recommended for COVID-19-negative patients according to 32% of respondents versus 10.7% for COVID-19-positive patients. A shorter fractionation was favoured for COVID-19-negative patients by 12% respondents versus 7.2% for COVID-19-positive patients.

Head and Neck Tumours

For patients with head and neck tumours, eligible for elective radiochemotherapy or RTH treatment, 87% of respondents suggested applying the standard protocol for COVID-19-negative patients. In comparison, 13% recommended a shorter fractionation schedule. No respondents were in favour of a delay in this group of patients. In COVID-19-positive patients, 35.3% of respondents suggested the standard RTH protocol, 14.7% favoured delaying the treatment, and 50% of respondents recommended a shorter fractionation.

Lung Tumours

Among patients with non-small cell lung cancer (NSCLC) eligible for lung stereotactic body radiotherapy (SBRT), 79.2% of respondents advised to apply the standard protocol for COVID-19-negative patients. In comparison, 12.5% of respondents suggested a shorter fractionation and 8.3% opted for a delay in RTH treatment. For COVID-19-positive patients, 28.6% of respondents recommended sticking to the standard RTH protocol, 52.4% favoured delaying the treatment, and 19% advised shortening the fractionation.

In patients with locally advanced NSCLC who were eligible for RTH treatment, the standard protocol was recommended by 82.6% of respondents for COVID-19-negative patients. In comparison, using the standard protocol was suggested by 26.1% of respondents for COVID-19-positive patients. A switch to a shorter fractionation was advised by 17.4% of respondents for COVID-19-negative patients and 26.1% for COVID-19-positive patients. A delay of the RTH treatment was advised in 47.8 % of respondents for COVID-19-positive patients. No respondents suggested a delay in the RTH treatment of COVID-19-negative patients.

Gastrointestinal Tumours

**Figure 2:**
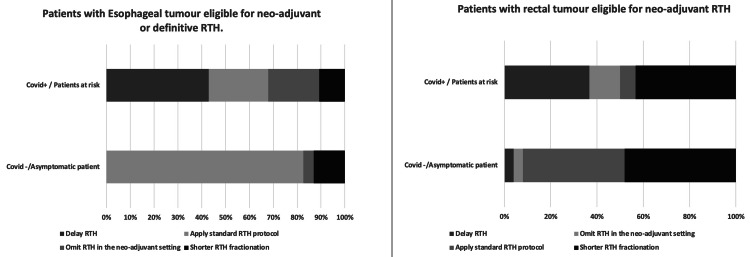
Adaptations of RTH treatments for esophageal tumours and rectal tumours according to the patient's COVID-19 status RTH: radiotherapy; COVID-19: coronavirus disease 2019

Figure 2 shows the RTH treatment adaptations in the case of gastrointestinal tumours. In patients with esophageal cancer eligible for neoadjuvant or definitive RTH, standard RTH was recommended by 82.6% of respondents for COVID-19-negative patients and 25% for COVID-19-positive patients. RTH treatment was not suggested in the case of neoadjuvant therapy in 4.3% of respondents for COVID-19-negative patients and 21.4% of respondents for COVID-19-positive patients.

RTH treatment delay was suggested by 42.9% of respondents for COVID-19-positive and by 0% of respondents for COVID-19-negative patients. A switch to shorter fractionation was suggested by 13% of respondents in COVID-19-negative patients and by 10.7% of respondents for COVID-19-positive patients.

In patients with rectal cancer eligible for neoadjuvant RTH alone or neoadjuvant or definitive radiochemotherapy (RTCT), 43.3% of respondents recommended using a shorter fractionation for COVID-19-positive patients versus 48% for COVID-19-negative patients. The institutional standard RTH protocol was advised by 44% of respondents for COVID-19-negative and 6.7% for COVID-19-positive patients. A delay in neoadjuvant RTH treatment was suggested by 4% of respondents for COVID-negative patients and 36.7 % for COVID-positive patients. A neoadjuvant RTH was not recommended by 4% of respondents for COVID-19-negative and 13.3% of respondents for COVID-19-positive patients.

Prostate Cancer

**Figure 3:**
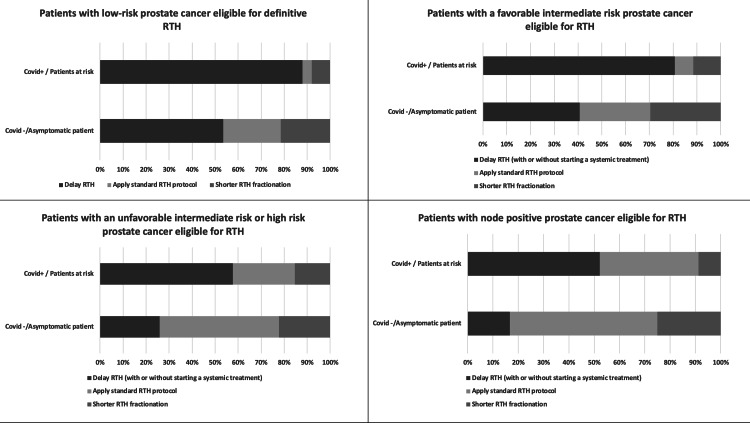
RTH treatment adaptations according to prostate cancer features and the patient's COVID-19 status RTH: radiotherapy; COVID-19: coronavirus disease 2019

Four conditions for patients with prostate cancer eligible for RTH treatment were considered. For patients with low-risk prostate cancer who were eligible for definitive RTH treatment, 53.6% of respondents favoured delaying RTH treatment for COVID-19-negative patients and 88% for COVID-19-positive patients. For COVID-19-negative patients, 21.4% of respondents advised switching to a shorter fractionation and 8% for COVID-positive patients. The standard protocol was proposed by 25% of respondents proposed for COVID-19-negative patients and by 4% for COVID-19-positive patients. This is shown in Figure 3.

For patients with intermediate-risk with a favourable prognosis who were eligible for definitive RTH, 40.7% of respondents recommended delaying RTH treatment for COVID-19-negative patients and 80.8% for COVID-19-positive patients. Almost one-third of respondents (29.6%) recommended switching to a shorter fractionation for COVID-19-negative patients, while 11.5% advised a shorter fractionation for COVID-19-negative patients. The standard protocol was recommended by 29.6% of respondents for COVID-19-negative patients and by 7.7% of respondents for COVID-19-positive patients.

For patients with intermediate-risk with an unfavourable prognosis who were eligible for definitive RTH, 25.9% of respondents recommended delaying RTH treatment for COVID-19-negative patients and 57.7% for COVID-19-positive patients. Respondents who advised shorter fractionation were 22.2% for COVID-19-negative patients and 15.4% for COVID-19-negative patients. The standard protocol was recommended by 51.9% of respondents for COVID-19-negative patients and 26.9% of respondents for COVID-19-positive patients.

For patients with prostate cancer and lymph node involvement who were eligible for RTH treatment, 16.7% of respondents advised delaying RTH treatment for COVID-19-negative patients. In comparison, a delayed treatment was recommended by 52.2% of respondents for COVID-19-positive patients. A shorter fractionation was proposed by 25% of respondents for COVID-19-negative patients and by 8.7% of respondents for COVID-19-negative patients. The standard protocol was recommended by 58.3 % of respondents for COVID-19-negative patients and 39.1% of COVID-19-positive patients.

Breast Cancer

**Figure 4:**
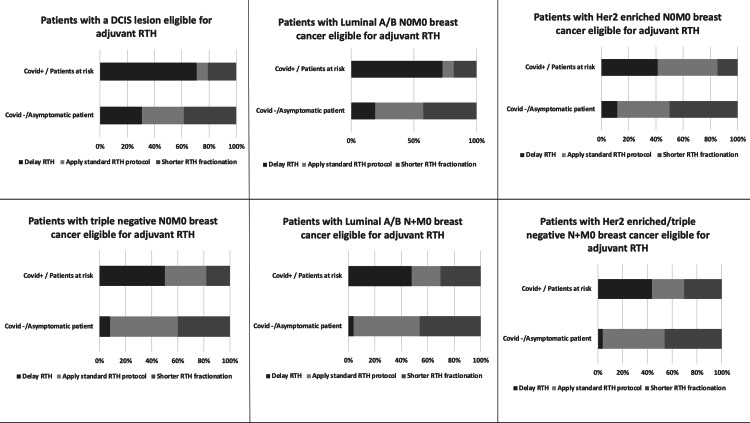
RTH treatment adaptation according to breast cancer features and the patient's COVID-19 status RTH: radiotherapy; DCIS: ductal carcinoma In situ; Her 2: human epidermal growth factor receptor 2; COVID-19: coronavirus disease 2019

Six conditions were analysed for breast cancer patients eligible for adjuvant RTH treatment. For patients with luminal A/B N0M0 breast cancer, eligible for adjuvant RTH treatment, almost 20% of respondents suggested delaying RTH for COVID-19-negative patients versus 72.7% for COVID-19-positive patients. Nearly 40% of respondents suggested applying the standard RTH treatment for COVID-19-negative patients versus 9.1% for COVID-19-positive patients. A shorter fractionation was recommended by 42.3% of respondents for COVID-19-negative patients versus 18.2% for COVID-19-positive patients (See Figure 4).

For patients with Her 2-enriched N0M0 breast cancer eligible for adjuvant RTH treatment, 11.5% of respondents favoured delaying RTH treatment for COVID-19-negative patients versus 41.2% for COVID-19-positive patients. Roughly 40% of respondents advised sticking to standard RTH treatment for COVID-19-negative patients versus 44.1% for COVID-19-positive patients. A shorter fractionation was recommended by 50% of respondents for COVID-19-negative patients versus 14.7% for COVID-19-positive patients.

For patients with triple-negative N0M0 breast cancer, eligible for adjuvant RTH, only 8% of respondents advised delaying RTH for COVID-19-negative patients versus 50% of COVID-19-positive patients. More than half of the respondents (52%) recommended standard RTH treatment for COVID-19-negative patients versus 31.8% for COVID-19-positive patients. Shorter fractionation was recommended by 40% of respondents for COVID-19-negative patients versus 18.2% for COVID-19-positive patients.

For patients with luminal A/B N+M0 breast cancer, eligible for adjuvant RTH treatment, 3.8% of respondents favoured delaying RTH treatment for COVID-19-negative patients versus 47.8% for COVID-19-positive patients. Half of the respondents advised following the standard RTH treatment for COVID-19-negative patients versus 21.7% for COVID-19-positive patients. Nearly half of the respondents (46.2% ) recommended switching to a shorter fractionation for COVID-19-negative patients versus 30.4% for COVID-19-positive patients.

For patients with Her 2-enriched or triple-negative N+M0 breast cancer, who are eligible for adjuvant RTH treatment, 3.8% of respondents advised delaying RTH treatment for COVID-19-negative patients versus 43.5% for COVID-19-positive patients. Half of the respondents (50%) recommended using the standard RTH for COVID-19-negative patients versus 26.1% for COVID-19-positive patients. A shorter fractionation was recommended by 46.2% of respondents for COVID-19-negative patients versus 30.4% for COVID-19-positive patients.

For patients with ductal carcinoma in situ (DCIS) lesions eligible for adjuvant RTH treatment, 30.8% of respondents favoured delaying RTH treatment for COVID-19-negative patients versus 70.8% for COVID-19-positive patients. Almost one-third of respondents (30.8%) advised using the standard RTH treatment for COVID-19-negative patients versus 8.3% of COVID-19-positive patients. A shorter fractionation was recommended by 38.5% of respondents for COVID-19-negative patients versus 20.8% for COVID-19-positive patients.

Brachytherapy

**Figure 5:**
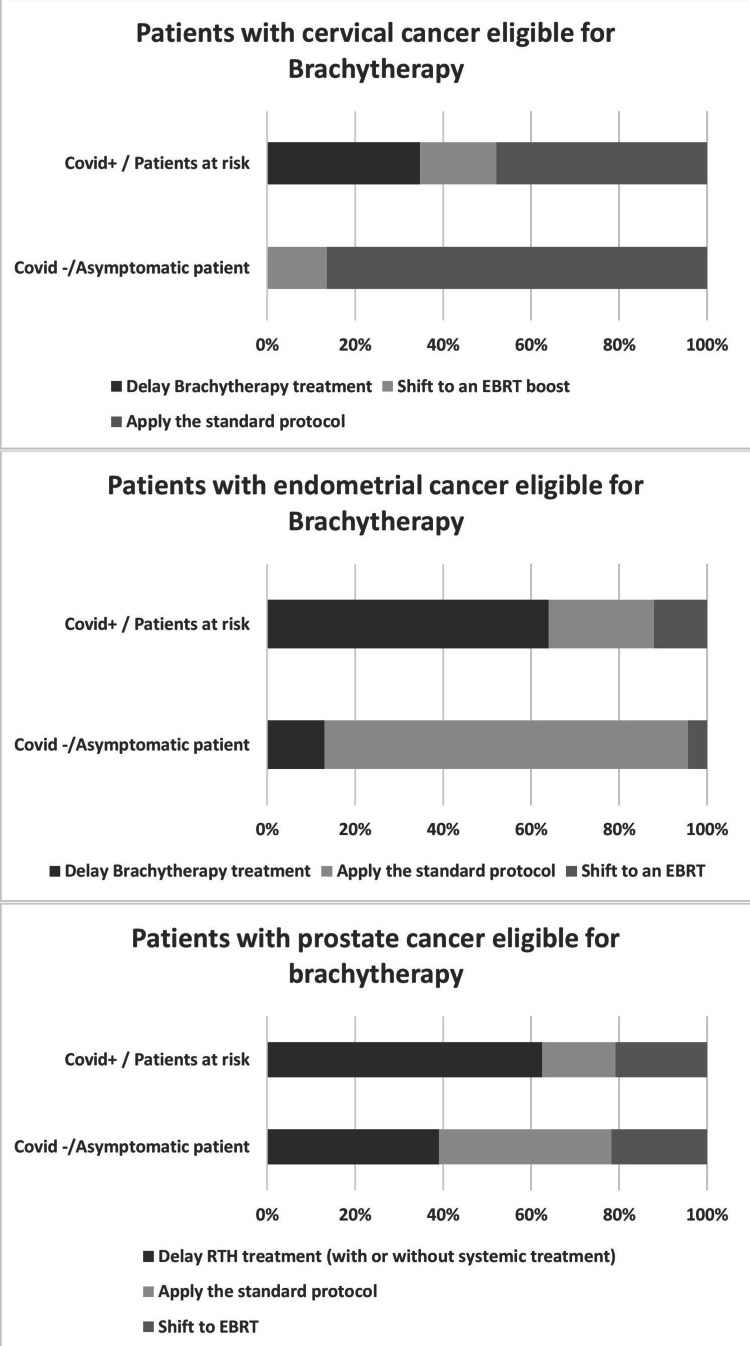
Brachytherapy treatment adaptations for different indications according to the patient's COVID-19 status RTH: radiotherapy; EBRT: external beam radiotherapy; COVID-19: coronavirus disease 2019

Respondents were asked how the COVID-19 pandemic changed brachytherapy treatments for cervical cancer, endometrial cancer, and prostate cancer. No respondent favoured delaying brachytherapy for patients with cervical cancer in COVID-19-negative patients versus 34% for COVID-19-positive patients. On the other hand, a switch to an EBRT boost was suggested by 13.6% of respondents for COVID-19-negative patients versus 17.4% for COVID-19-positive patients. Most of the respondents (86.4%) preferred the standard brachytherapy protocol for COVID-19-negative patients versus 47.8% for COVID-19-positive patients (See Figure 5).

In the case of a patient presenting with endometrial cancer that was eligible for brachytherapy, 82.6% of respondents suggested sticking to the standard protocol for COVID-19-negative patients versus 24% for COVID-19-positive patients. Only 13% of respondents advised delaying RTH treatment for COVID-19-negative patients versus 64% for COVID-19-positive patients. A small number of respondents (4.3%) recommended switching to an EBRT boost for COVID-19-negative versus 12% for COVID-19-positive patients.

In patients with prostate cancer eligible for brachytherapy treatment, 39.1% of respondents recommended the standard brachytherapy protocol for COVID-19-negative patients versus 16% for COVID-19-positive patients. Almost 40% (39.1% ) of respondents favoured delaying RTH treatment for COVID-19-negative patients versus 62.5% of COVID-19-positive patients. A fifth of respondents (21.7% ) advised switching to EBRT for COVID-19-negative and 20% for COVID-19-positive patients.

Palliative radiotherapy

For patients with spinal cord compression, standard RTH treatment was intended to be maintained in 51.72% of respondents for COVID-19-negative versus 23.53% for COVID-19-positive patients. Of the respondents, 7% favoured both alternative treatments without RTH or best supportive care without RTH for COVID-19-negative patients. For COVID-19-positive patients, 20.6% of respondents were in favour of alternative treatments without RTH and 14.7% suggested best supportive care without RTH.

Regardless of location, for patients having painful metastasis, 40% of respondents advised sticking to the standard RTH treatment protocol for COVID-19-negative patients, compared to 12.9% for COVID-19-positive patients. A shorter fractionation scheme was favoured by respondents for both groups as follows: 34.3% in COVID-19-negative and 38.7% in COVID-19-positive patients.

Respondents favoured alternative treatment without RTH or best supportive care without RTH in 17.1% and 8.6%, respectively, for COVID-19-negative patients and 29.6% and 25.9%, respectively, for COVID-19-positive patients.

## Discussion

Due to the COVID-19 pandemic, European RTH departments had to adapt their treatment schedule to cope with the first wave in March 2020. Early after the pandemic outbreak, some recommendations were issued by national or international organizations or institutions to help ROs adapt RTH treatments [5-15] when they would face a decrease in their resources due to the pandemic.

Slotman et al. surveyed 139 RTH departments in Europe and reported on the significant adaptations in the management of COVID-19 [4]. The authors showed that a substantial delay in RTH treatment was adopted for prostate cancer (low risk 62%; intermediate risk 40%, high risk 20%), non-malignant indications (38%), early breast cancer (31%), and palliative non-malignant indications (25%), non-melanoma skin tumours (16%), low-grade gliomas (16%) and SBRT for oligometastatic disease (10%). These results are consistent with those of the present survey. However, RTH treatment indications in Slotman et al. [4] did not correlate with patient COVID-19 status as in the current study. In addition, respondents in our study were asked to suggest which RTH treatments should have been postponed and which treatments should have been discontinued or shortened by hypofractionation.

The present study has yielded some interesting results: First, although we included only a small number of respondents compared to Slotman et al. [4], we observed the same general trends in the postponement of treatments tailored to different cancer types and stages. This congruence suggests that in future surveys, even a limited sample of facilities could quickly provide an overview of the heuristic ROs to respond to the rapid shortage of resources for RTH treatment.

Second, for COVID-19-negative patients, most respondents recommended adherence to RTH treatment recommendations. In fact, for several protocols, respondents suggested no adjustment to RTH treatment protocols, including the protocol for spinal cord compression, glioblastoma, low-grade glioma, lung tumours, digestive tract cancers, prostate tumours, and breast tumours.

However, in COVID-19-positive patients, a significant proportion of respondents suggested delaying RTH treatment for indications such as spinal cord compression, glioblastoma, high-grade glioma, and lung cancer even if the delay was not recommended in the literature. This result shows how the COVID-19 status of the patient can influence ROs' decision-making, even when the RTH treatment indication is urgent.

Third, when hypofractionated protocols were available in the literature, as in the case of palliative radiotherapy [15], breast cancer [6,7], glioblastoma [11], and rectal cancer [12], these shorter fractionation schemes were recommended much more frequently during the pandemic in the COVID-19-positive patient group than in the COVID-19-negative group. In these clinical settings, the COVID-19 status of the patient might have been considered in the decision-making dilemma overwhelming ROs facing the pandemic.

Fourth, for head and neck tumours, only 14.7% of respondents opted to delay radiotherapy in COVID-19-positive patients. This percentage is lower than for other cancers, and this may be related to the presence of specific recommendations considering the COVID-19 status [13].

Finally, many respondents recommended delaying brachytherapy treatment in COVID-19-positive patients for cervical and endometrial cancer. However, the delay was not in line with published recommendations, which advised against changing the timing of brachytherapy due to the pandemic [14].

Limitations

We acknowledge that our study has three significant limitations. Even though the respondents represent many countries in Europe, the limited number of respondents may have influenced our results. However, in the sample of respondents, the trends captured in this study are similar within the different countries. The results collected should be confirmed in a larger sample of academic institutions.

Another limitation is that most recommendations were published within the first two weeks of April 2020, raising the possibility that not all respondents were aware of the rapidly emerging literature during the first wave of the pandemic crisis. Finally, the present survey was sent to academic respondents. The trends captured in this survey may not reflect trends in other centres with different organisations, sizes, and facilities.

## Conclusions

The COVID-19 status of patients had a significant impact on RTH treatment decision-making, regardless of cancer type and aggressiveness in different RTH departments in Europe. Standard institutional protocols or published recommendations for radiotherapy during the Covid-19 pandemic were mainly followed in asymptomatic patients or those with negative COVID-19 status. This was less the case for patients with a positive COVID-19 status or patients suspected to be COVID-19 positive. This highlights the need for recommendations not only by cancer type but also by taking into account the COVID-19 status of patients.

## Figures and Tables

**Figure 6:**
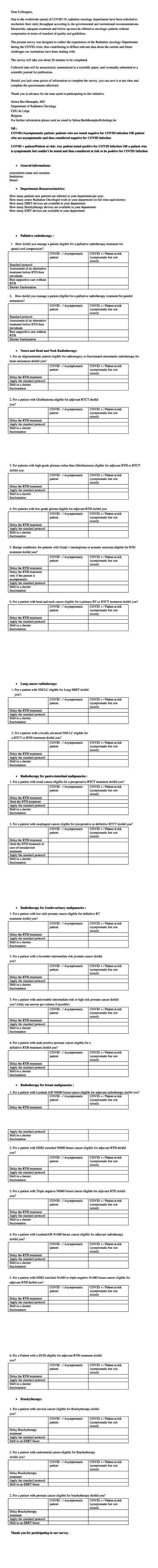
The survey COVID-19: coronavirus disease 2019; EBRT: external beam radiotherapy; IORT: intraoperative radiotherapy; RTH: radiotherapy; RTCT: radiotherapy plus chemotherapy; NSCLC: non-small cell lung cancer
